# Anti-fatigue activity of dripped spent hens chicken essence in ICR mice

**DOI:** 10.5713/ab.22.0172

**Published:** 2022-09-02

**Authors:** Ti Chun Chang, Wei Cheng Chen, Chao Wei Huang, Liang Chuan Lin, Jen Shinn Lin, Fu Yuan Cheng

**Affiliations:** 1 Department of Food Science, National Pingtung University of Science and Technology, Pingtung 912301, Taiwan; 2 Department of Biomechatronics Engineering, National Pingtung University of Science and Technology, Pingtung 912301, Taiwan; 3 Department of Tropical Agriculture and International Cooperation, National Pingtung University of Science and Technology, Pingtung 912301, Taiwan; 4 Department of Animal Science, National Chung Hsin University, Taichung 40227, Taiwan; 5 Department of Animal Science, National Pingtung University of Science and Technology, Pingtung 912301, Taiwan

**Keywords:** Anti-fatigue, Branched Chain Amino Acids, Chicken Essence, Spent Hen

## Abstract

**Objective:**

Chicken essence and branched chain amino acid (BCAA) supplementation has been recognized to significantly relieve fatigue. To obtain chicken essence with high amounts of BCAA, spent hens herein was used to prepare dripped chicken essence (SCE) and compared with commercial dripped chicken essence (CCE) for *in vivo* anti-fatigue effect.

**Methods:**

To determine the effect on anti-fatigue by dripped chicken essence, the exhaustive swimming was performed. Thirty-two 7-week ICR mice were divided into four groups, which included the control group (CG), CCE, SCE-1X and SCE-2X. The mice were given daily oral administration (0.012 mL/g body weight/d). The fatigue index analysis was conducted weekly.

**Results:**

The results showed that SCE had a higher BCAA level as expected, and mice treated with dripped chicken essence (CCE and SCE) could significantly improve exercise performance. The lower blood lactate level, blood urea nitrogen level and creatine phosphokinase activity were found in the supplement of SCE group compared with the CCE group, which suggested that the SCE possessed strong anti-fatigue ability. This could possibly be due to the higher content of BCAA.

**Conclusion:**

In this study, SCE promoted recovery from physical fatigue in mice and elevated endurance ability. Among them, the double dose (SCE-2X) showed the strongest anti-fatigue ability. Taken together, spent chickens could be a good source of chicken essence to improve the effect of anti-fatigue.

## INTRODUCTION

Chicken essence has abundant of nutrition, including protein, peptides, amino acid, free fatty acid (FFA), vitamins, minerals, trace elements, which can be a liquid nutritional supplement for humans. Chicken essence is a functional food prepared by boiling chickens in water under high pressure-high temperature processing to transfer the long-chain proteins of chickens into short-chain proteins or peptides. It is a common nutrient supplement in many Asian countries. It has been recognized that chicken essence has functional effects such as anti-fatigue, anti-stress, and stabilizing blood glucose to improve performance during the exercise [1,2]. Studies have shown that chicken essence extracted from whole chickens by long-term heating presented special nutrients with a significant anti-fatigue effect in mice. Additionally, it can effectively enhance the elimination of lactic acid accumulated in the blood, and reduce the concentration of creatine phosphokinase (CPK) and blood ammonia, thereby decreasing fatigue caused by exercising [3,4].

Branched chain amino acids (BCAA), including leucine, isoleucine and valine have all been shown to improve mental fatigue and physical performance responses to training and performance [5]. Similar results were also stated by Wiśnik et al [6] who indicated that soccer players with BCAA supplementation had better psychomotor performance. Nowadays, chicken essence is considered as a nutritional supplement rich in BCAA with certain inhibitory effect on the occurrence of fatigue [3]. However, there are large amounts of free amino acid in chicken essence, some of which are recognized as bitter tastes such as imidazole dipeptides (carnosine and anserine), arginine, histidine, and methionine [7,8]. It has revealed that some proline-based diketopiperazines with impart a metallic bitter taste were quantified in commercial chicken essence before and after thermal treatment at 130°C for 1 h [9]. Contrary to chicken essence, the dripped chicken essence is produced using chicken in a long time steaming extraction (about 100°C for 10 h) to convert chicken proteins into smaller peptides and amino acids. It’s taste is similar to that of a chicken soup, which makes it more acceptable to consumers.

It is well known that the composition of amino acids in chicken breast meat is influenced by daily feed and ages. In particular, the BCAA content in chicken meat increases with age [10]. Spent hens have a high content of BCAA and collagen, which are major by-products of the egg industry [11]. Using the spent hens for further application could add a new value to the by-product. The use of spent-hen for further application could add a new or more value to these by-products. However, the collagen content increases with age, causing the spent hen difficult to process. Therefore, in this study, the spent hens were prepared as dripped chicken essence and compared in amino acid composition with commercial dripped chicken essence (prepared from Taiwan native chicken, 21 weeks old) that has already approved anti-fatigue healthy food certification in Taiwan. The *in vivo* anti-fatigue effects, including exhaustive swimming time, blood biochemical indicators and liver glycogen analysis, were also conducted to evaluate a potential candidate for the preparation of chicken essence from spent hens.

## MATERIALS AND METHODS

### Preparation of dripped chicken essence

Twenty-four frozen (storage at −20°C) carcasses of 85 to 100 weeks spent hens were purchased from a commercial meat factory (Yunlin, Taiwan). They were thawed in a refrigerator at 4°C for 24 h, washed, and the heads, necks and fat were all removed before cutting into eight pieces, individually. The samples were placed in a vessel covered with a lid and boiled with steam at 100°C in a cauldron for 10 h. After extraction, the dripped chicken essence was then sieved, defatted, packed and stored at −80°C for further analysis. For obtaining high concentration of spent hens dripped chicken essence (SCE-2X), the collecting SCE sample was condensed by vacuum concentration to twice the concentration. The commercial dripped chicken essence approved with anti-fatigue healthy food certification (No. A00222; TFDA, Taipei, Taiwan) was purchased from a market (Tainan, Tainan, Taiwan) and used as a positive control.

### Amino acids composition analysis

Amino acid composition was analyzed with a reference to Simpson et al [12] with minor modifications. The samples were hydrolyzed with 4 N methanesulfonic acid containing 0.2% tryptamine at 115°C under vacuum for 22 to 72 h. Then, the hydrolysates were neutralized by 3.5 M NaOH and quantified amino acids using automatic amino acid analyzer (model 6300; Beckman, Duarte, CA, USA).

### Evaluation of *in vivo* anti-fatigue

According to Huang et al [3], thirty-two 4 weeks male Institute of Cancer Research (ICR) mice were purchased from BioLASCO Taiwan Co., Ltd. (Taipei, Taiwan) and housed in a room with air conditions (25°C±2°C in ambient temperature, 60%±10% in relative humidity, and 12 h light-dark cycle with lighting from 7:00 to 19:00). A laboratory diet (MFG; Oriental Yeast, Tokyo, Japan) and distilled water was available *ad libitum* throughout the experiment. After a week of acclimation, 32 ICR mice were randomly divided into 4 groups: control group (CG), commercial dripped chicken essence as positive control (CCE), and two dosages of spent hens dripped chicken essence (SCE and SCE-2X). The daily recommended dose of dripped chicken essence for humans is 60 mL per day. Based on the conversion coefficient in body surface area between mice and humans (12.3) [13], 1.2 mL/100 g body weight (BW)/d was orally administered to mice by using a gavage feeding needle in 7 weeks of age. The same volume of water replaced sample for control group (CG). The fatigue index analysis was conducted weekly for 4 weeks. At the end of the experiment, the tissues and blood were collected for further analysis. All experimental procedures involving animals and protocols used in this study were reviewed and approved by the Institutional Animal Care and Use Committee of National Chung Hsing University, Taichung, Taiwan. The IACUC approval No. was 105-007.

### Exhaustive swimming test

Exhaustive swimming test was carried out according to the method of Bao et al [14]. Briefly, the mice were fasted for 16 h and orally administered with sample or water 30 min before testing. From each group, 8 mice were taken out and loaded on the back with lead wire (3% of body weight), placed into a water pool (diameter of 15 cm, depth of 20 cm, temperature of water of 27°C±1°C) and then recorded the time from swimming to exhaustion based on their heads sunk into water for 8 seconds. Blood samples were collected by tail vein bleeding at the beginning, after swimming to exhaustion and 30 min after being allowed to rest. Blood glucose and lactate was analyzed by blood glucose meter (Roche Diagnostics GmbH, Mannheim, Germany) and lactate test meter (Arkray Factory, Inc., Shiga, Japan), respectively.

### Analysis of serum biochemical parameters

The procedure was modified according to Qi et al [15]. During the final treatment with sample or water, the mice were allowed to rest for 30 min. 8 mice were taken from each group and sacrificed with isoflurane 10 min after being forced to swim without back loads. The blood samples were collected by cardiac puncture and centrifuged at 3,000 rpm at 4°C for 10 min. The separated serum was collected and stored at −80°C until analyzed for blood urea nitrogen (BUN) and CPK by blood auto-analyzer (CIBA-Corning, Missouri, TX, USA).

### Statistical analysis

Data were presented as means±standard deviation. Analysis of variance was conducted using one-way analysis of variance followed by Duncan’s multiple range tests by IBM SPSS statistical package (version 20.0). Differences were considered significant at the level of p<0.05.

## RESULTS AND DISCUSSION

### Amino acids composition of dripped chicken essence

There was a higher total amount of amino acid in SCE (4,057.5 mg/100 g) than CCE (3,157.2 mg/100 g). Higher contents of tyrosine (1.3%), tryptophan (1.58%), lysine (4.8%), histidine (5.56), which were related to bioactive peptides, were found in SCE and not in CCE. In addition, the total amount and percentage of BCAA (387.4 mg/100 g, 9.70%), essential amino acids (EAA) (1,420.3 mg/100 g, 35.00 %), positively charged amino acids (905.4 mg/100 g, 22.31%), negative charged amino acids (998.9 mg/100 g, 24.62%) and aromatic amino acids (205.1 mg/100 g, 5.05%) were higher in SCE, whereas hydrophobic amino acids (35.05%) were higher in CCE (Table 1). Incidentally, dripped chicken essence was lighter in color than traditional chicken essence and scored higher in flavor, taste and overall acceptability in sensory evaluation (data not shown). Wu and Shiau [8] indicated that the hydrolyzed amino acids were constituent amino acids of low molecular weight peptides in chicken essence among which glycine, proline, alanine, glutamic acid and aspartic was predominant in small peptides. Li et al [16] reported that black bean peptide contained 11.02% glutamic acid and 6.83% aspartic acid, which could possibly help in delaying the occurrence of fatigue owed to glutamic acid which improves the regulation of the nervous system and aspartic acid, thus reducing the blood ammonia concentration. Moreover, as previously stated, BCAA contributes to various physiological effects during exercise. It is the only metabolizable amino acid in skeletal muscle, energy substrates during exercise and is involved in protein synthesis with acting as a precursor for other amino acid synthesis [5,17]. This finding supports our results that SCE could be a better source to improve the effect of anti-fatigue with better nutritional value than CCE due to the higher amount and percentage of EAA and bioactive related amino acids in SCE.

### Exhaustive swimming time

**Figure 1:**
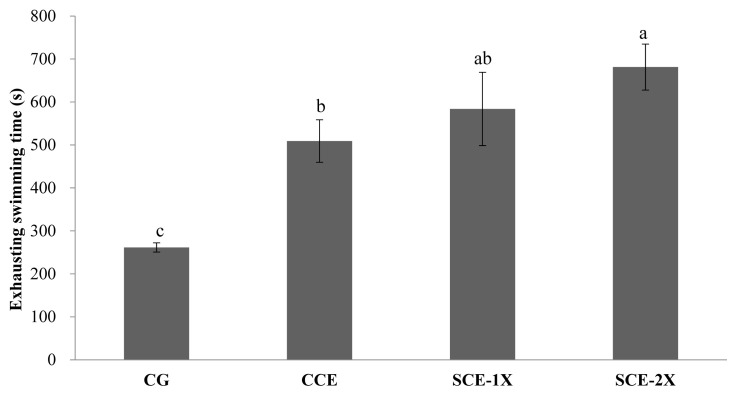
The effect of different treatments on exhausting swimming time in mice. Values are means±standard deviation for n = 8 mice per group. CG, control group; CCE, commercial dripped chicken essence; SCE-1X, spent hen dripped chicken essence; SCE-2X, double dosage of spent hen dripped chicken essence. ^a–c^ Bars with different letters are significantly different at p<0.05.

The exhaustive swimming time of mice in each group, which is a CG group (261.5 s), CCE (509.13 s), SCE-1X (583.75 s), and SCE-2X (681.25 s), respectively (Figure 1). It shows that chicken essence could prolong the duration significantly (p<0.05). The exhaustive swimming test is often used as an evaluation of *in vivo* anti-fatigue, in which the swimming time indicates the degree of anti-fatigue in the animal. An increase in exercise tolerance can be used to represent the anti-fatigue activity. The longer swimming time interprets the reduced susceptibility to fatigue [3,18]. Several studies have indicated that 5-hydroxytryptamine could be increased in synthesis during long-term physical exercise and leaded to weaken the central nervous system that resulted in central fatigue [19]. Most of the amino acids in particular BCAA in the plasma, decreased rapidly when the exercise time was prolonged. BCAA was not absorbed by the liver after intake, but rapidly increased in the plasma that contributed in providing a balance of FFA and inhibited the increasing ratio of free tryptophan/BCAA, which benefited the delay of fatigue while improving endurance [20]. AbuMoh’d et al [21] discussed the effects of oral BCAA intake on central fatigue by using a double-blind and placebo-controlled of long-distance runners. Results showed that the intake of oral BCAA supplements one hour before incremental exercise increased the oxidation of BCAA, thereby reducing plasma levels of FFA and serotonin, which prolonged the endurance of long-distant runners. Similar results were also observed in this study. All dripped chicken essence supplements improved the endurance of mice, reduced fatigue, and effectively prolonged exhaustive swimming time, while SCE-2X showed the highest activity that appeared to be related to the highest BCAA content.

### Blood glucose

**Figure 2:**
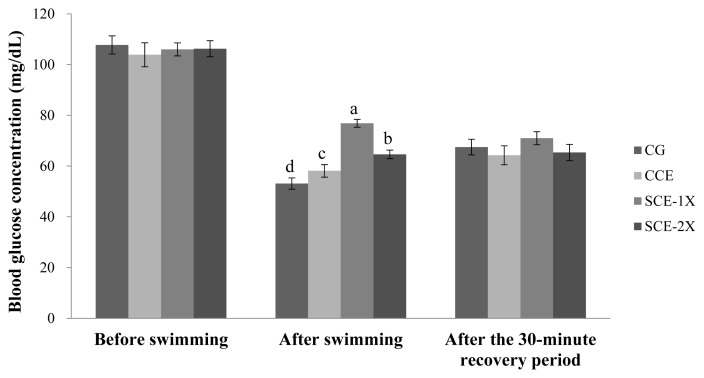
The effect of different treatments on the blood glucose concentration in mice. Values are means±standard deviation for n = 8 mice per group. CG, control group; CCE, commercial dripped chicken essence; SCE-1X, spent hen dripped chicken essence; SCE-2X, double dosage of spent hen dripped chicken essence. ^a–d^ Bars with different letters are significantly different at p<0.05.

There were no significant differences in blood glucose in all mice before the exhaustive swimming test. After the test, the blood glucose of all mice was reduced and the significantly higher glucose contents were observed in the SCE-1X (76.88 mg/dL), SCE-2X (64.63 mg/dL), and CCE groups (58.13 mg/dL) compared with the CG group (53.13 mg/dL) (p< 0.05) (Figure 2). During prolonged exercise, the physiological regulation would maintain the blood glucose level. The lack of blood glucose would enhance the metabolism of plasma FFA that induced fatigue and reduced exercise performance [22]. Assenza et al [23] reported that supplement of BCAA conduced to prevent deterioration of physical performance, which usually resulted from a lack of muscle glycogen after endurance exercises. It was also indicated that the intake of BCAA could improve exercise performance and slightly reduced the decrease in blood glucose level during exercising. However, Kim et al [24] suggested that the intake of BCAA had lower blood glucose level than the placebo group at all exercising test times because of less secretion of a central fatigue substance such as serotonin, which enhanced exercise performance with greater consumption of energy. Inconsist results were exhibited in this study. The supplement of dripped chicken essence groups seemed to be complement of glucose that maintained the blood glucose level compared with the control group. In particular SCE group with the higher BCAA content might be more helpful to the supply of energy and blood glucose metabolism efficiency.

### Blood lactate

**Figure 3:**
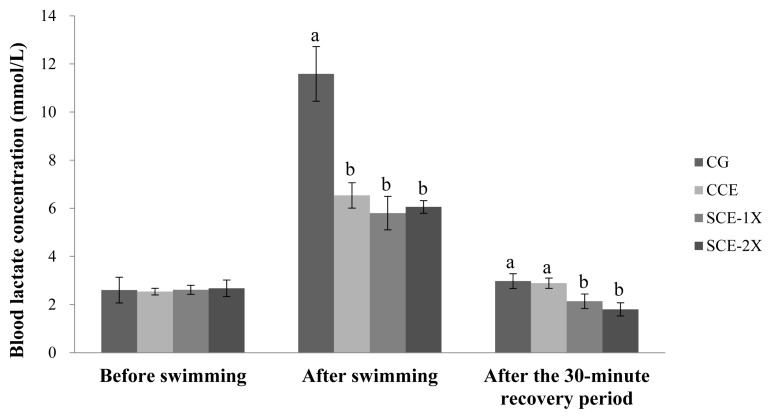
The effect of different treatments on the blood lactate concentration in mice. Values are means±standard deviation for n = 8 mice per group. CG, control group; CCE, commercial dripped chicken essence; SCE-1X, spent hen dripped chicken essence; SCE-2X, double dosage of spent hen dripped chicken essence. ^a,b^ Bars with different letters are significantly different at p<0.05.

The blood lactate had no significant difference among the groups before the swimming test. After the exhaustive swimming test, the blood lactate concentrations were increased, while the CG group had a significant higher value (11.59 mmol/L) than that of CCE (6.54 mmol/L), SCE-1X (5.80 mmol/L) and SCE-2X (6.06 mmol/L) (p<0.05). After the mice had rested for 30 min, the blood lactates of all groups were scavenged and recovered to basal levels. Meanwhile, the lower blood lactate was found in both the SCE-1X and SCE-2X groups compared with the CG and CCE groups (p<0.05) (Figure 3). The lactate accumulation is regarded as a result of anaerobic energy production and it is often used as an indicator of fatigue. During intensive exercise, glycolysis of carbohydrates under anaerobic conditions provided energy mainly for muscle contraction that caused an increase of lactic acid and lowered intramuscular pH value. In some cases, the concentration of intracellular lactic acid could reach 30 mM and the intracellular pH dripped to the range of 6.2 to 6.5 [25]. Fitts [26] indicated that a low pH value in muscle would bring fatigue, which reduces 30% to 35% activity and endurance of muscle contraction. Therefore, diminishing accumulation of lactate or scavenging would be of a great benefit for anti-fatigue. Monteiro et al [27] reported that BCAA supplementation attenuated the lactate production in pregnant rats, probably by increasing its utilization as an alternative energy substrate in trained animals. This was in agreement with our results in which the supplement of dripped chicken essence (CCE, SCE-1X, and SCE-2X) demonstrated that the attenuation of lactate production might be due to BCAA content within.

### Blood urea nitrogen and creatine phosphokinase

**Figure 4:**
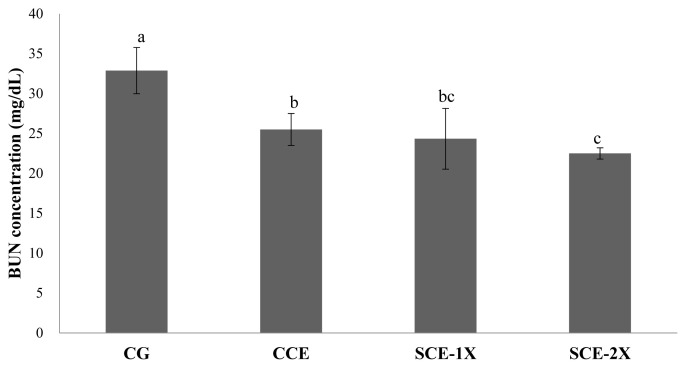
The blood urea nitrogen concentration of different treatments in mice. Values are means±standard deviation for n = 8 mice per group. ^a–c^ Bars with different letters are significantly different at p<0.05. CG, control group; CCE, commercial dripped chicken essence; SCE-1X, spent hen dripped chicken essence; SCE-2X, double dosage of spent hen dripped chicken essence.

**Figure 5:**
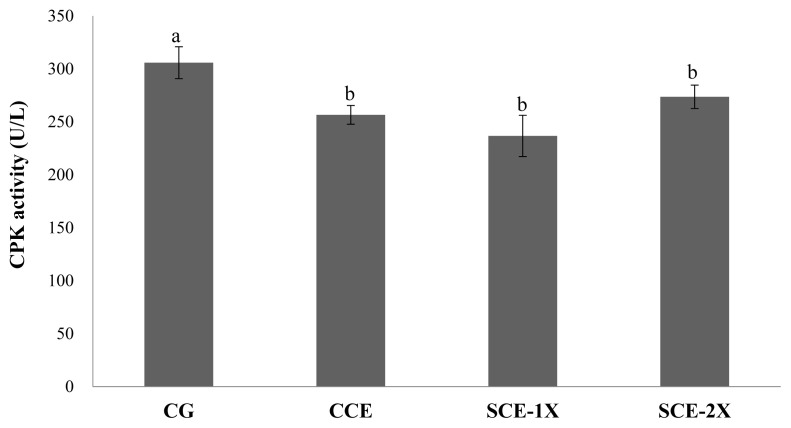
The creatine phosphokinase activity of different treatments in mice. Values are means±standard deviation for n = 8 mice per group. ^a,b^ Bars with different letters are significantly different at p<0.05. CG, control group; CCE, commercial dripped chicken essence; SCE-1X, spent hen dripped chicken essence; SCE-2X, double dosage of spent hen dripped chicken essence.

Supplementation of dripped chicken essence induced significant decreases in blood BUN for SCE-1X (24.33 mg/dL), SCE-2X (22.50 mg/dL), and CCE (25.50 mg/dL) compared with CG (32.88 mg/dL) (p<0.05). Similar results were obtained in CPK activity in which the CG group had the significantly lower CPK activity (305.88 U/L) than that of CCE (256.63 U/L), SCE-1X (236.75 U/L), and SCE-2X (273.67 U/L) (p< 0.05) (Figure 4). The BUN is a metabolite from protein and amino acid degradation. Urea is formed in the liver and transported through the blood to the kidneys for excretion. The concentration of urea nitrogen in the blood can be used as an indicator of the state of the kidneys, because it is completely responsible for removing urea from the blood and the higher BUN can be observed under stress and fatigue [18]. Consistent results indicated that BUN was found to increase significantly after exercise, which could reveal the degree of physical fatigue [28]. BUN could be a sensitive index of physical fatigue status, it was also reported that BUN was negatively correlated with exercise tolerance and a higher BUN could be found in poor exercise endurance [29]. Results of this study showed that the supplementation of CCE, SCE-1X, and SCE-2X could effectively lower protein degradation and energy metabolism, lower the accumulation of BUN, and improve the endurance ability of mice during exercise (Figure 5).

It has been recognized that intensity exercise may induce oxidative stress, such as producing reactive oxygen species and free radicals, which can lead to inflammation, lipid peroxidation, damage to membrane permeability and cell tissues, and cause cell or tissue damage. Meanwhile, CPK would be released into serum, which could therefore often be used as a parameter for tissue cell damage [30]. In the past, BCAA has been reported to be the major contributor to anti-fatigue during exercise. Coombes and McNaughton [5] demonstrated that supplementation of BCAA reduced the muscle damage associated with endurance exercise and might have a sustained effect on lower levels of intramuscular catabolism within a few days after exercise. Huang et al [1] reported that chicken essence possessed an attenuation of fatigue with lowering blood lactate, BUN and CPK because of the high content of free amino acids within. This was consistent with our observations in which dripped chicken essence diminished the BUN levels and CPK activity in mice. Similar to the results of Li et al [16], SCE-2X showed the best inhibitory activity, and this might be attributable to its highest BCAA content.

## CONCLUSION

The present study suggested that a higher content of amino acids and BCAA was obtained in SCE compared with the CCE. SCE indeed elevated endurance ability, promoting recovery from physical fatigue as the same as the effect of CCE in mice. The lower BUN concentration and CPK activity in both SCE and CCE indicated the prevention of cellular damage in mice. Additionally, increased double dose (SCE-2X) exhibited stronger effects, which suggested that the beneficial an effect of SCE was dose-dependent. This study provides the potential value of spent hens prepared as a chicken essence.

## Figures and Tables

**Table 1:** Amino acid composition of spent hens and commercial dripped chicken essence

Item	Dripped chicken essence			

	Spent hens		Commercial	
	
	(%)	(mg/100 g)	(%)	(mg/100 g)
Amino acid				
Valine	2.83	114.8	2.56	80.7
Isoleucine	2.01	81.6	1.83	57.8
Leucine	4.71	191.0	3.78	119.3
Phenylalanine	2.17	88.2	2.35	74.1
Cystine	0.48	19.5	0.56	17.8
Arginine	7.12	288.7	9.21	290.8
Proline	5.52	223.9	12.76	402.8
Glutamic acid	17.28	701.0	13.91	439.3
Histidine	5.56	225.5	4.00	126.4
Methionine	0.63	25.4	1.11	34.9
Lysine	6.32	256.3	4.80	151.7
Tyrosine	1.30	52.9	0.87	27.6
Aspartic Acid	7.34	297.9	6.16	194.5
Serine	3.39	137.6	3.13	98.9
Glycine	19.24	780.8	21.25	670.8
Threonine	2.09	84.8	2.47	78.1
Alanine	10.44	423.6	9.12	287.9
Tryptophan	1.58	64.0	0.12	3.8
Total	100.01	4,057.5	100.00	3,157.2
BCAA	9.70	387.4	8.17	257.8
EAA	35.00	1,420.3	32.23	1,017.6
HAA	31.67	1,284.9	35.05	1,106.7
PCAA	22.31	905.4	17.93	566.0
NCAA	24.62	998.9	20.07	633.8
AAA	5.05	205.1	3.34	105.5

BCAA, branched chain amino acid; EAA, essential amino acid; HAA, combined total of hydrophobic amino acids including cysteine, alanine, tyrosine, valine, methionine, tryptophan, phenylalanine, isoleucine, leucine, and proline; PCAA, positively charged amino acids (alanine, histidine, and lysine); NCAA, negative charged amino acids (aspartic acid and glutamic acid); AAA, aromatic amino acid (phenylalanine, tryptophan, and tyrosine).
